# Molecular Modeling of Signal Peptide Recognition by Eukaryotic Sec Complexes

**DOI:** 10.3390/ijms221910705

**Published:** 2021-10-02

**Authors:** Pratiti Bhadra, Volkhard Helms

**Affiliations:** Center for Bioinformatics, Saarland Informatics Campus, Saarland University, Postfach 15 11 50, 66041 Saarbruecken, Germany; pratiti.bhadra@bioinformatik.uni-saarland.de

**Keywords:** signal peptide, Sec61 complex, protein translocation, nascent peptide chain, membrane insertion, molecular modelling, molecular dynamics simulations, molecular docking

## Abstract

Here, we review recent molecular modelling and simulation studies of the Sec translocon, the primary component/channel of protein translocation into the endoplasmic reticulum (ER) and bacterial periplasm, respectively. Our focus is placed on the eukaryotic Sec61, but we also mention modelling studies on prokaryotic SecY since both systems operate in related ways. Cryo-EM structures are now available for different conformational states of the Sec61 complex, ranging from the idle or closed state over an inhibited state with the inhibitor mycolactone bound near the lateral gate, up to a translocating state with bound substrate peptide in the translocation pore. For all these states, computational studies have addressed the conformational dynamics of the translocon with respect to the pore ring, the plug region, and the lateral gate. Also, molecular simulations are addressing mechanistic issues of insertion into the ER membrane vs. translocation into the ER, how signal-peptides are recognised at all in the translocation pore, and how accessory proteins affect the Sec61 conformation in the co- and post-translational pathways.

## 1. Introduction

In eukaryotes, the majority of protein biosynthesis is carried out either by cytosolic ribosomes or by ribosomes that are attached to the ER membrane [1,2]. In the latter case, the newly synthesised “nascent polypeptide chain” (NC) is typically either translocated into the ER or laterally inserted into the ER membrane via an integral membrane protein complex termed translocon (with the exception of tail-anchored membrane proteins). In eukaryotes, the central component of this integral membrane protein complex is termed Sec61 complex. Prokaryotes also possess Sec channels named SecYEG which are responsible for protein secretion into the periplasm of the cell and for inserting membrane proteins into the cell membrane.

Proteins that should be translocated across the ER membrane typically carry a “signal peptide” (SP) at their N-terminus. In the ER, this sequence is then cleaved off by the enzyme complex signal peptidase. Alternatively, membrane proteins devoid of a SP are recognised by their first transmembrane (TM) helix. In both cases, a lateral gate opens in the translocation pore and either the TM portions of these proteins or the cleaved SP exits laterally into the lipid bilayer. In the latter case, the cleaved peptide chain is released into the ER interior. Here, we will concentrate on the translocation through Sec pores.

**Figure 1:**
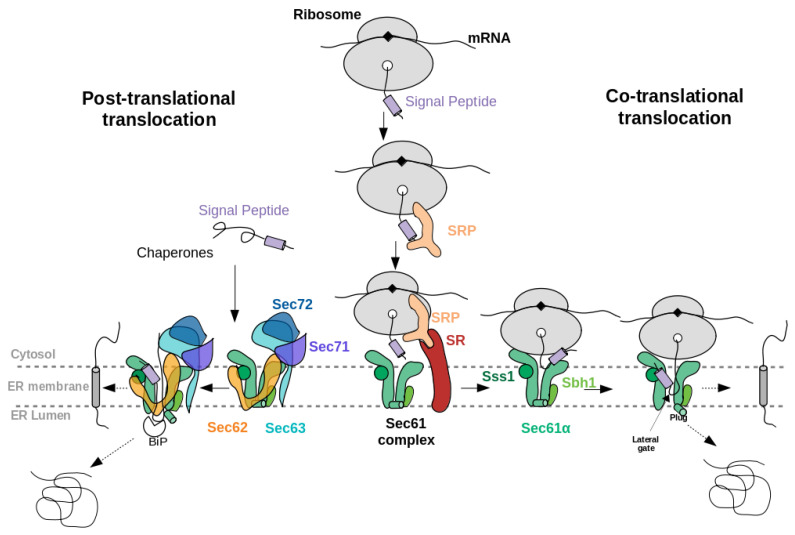
Schematic illustration of (left) the post-translational and (right) co-translational translocation of a eukaryotic secretory protein through the Sec61 complex. In both co- and post-translational pathways, accessory proteins such as Sec62 and Sec63 help in the translocation of precursor proteins with “weak” signal peptides (see section “properties of signal peptides”). The accessory proteins Sec71 and Sec72 exist only in yeast. Sbh1 and Sss1 are the name of the β and γ sub-units of yeast Sec61 complex.

When a newly synthesised NC exits from the ribosome, often a protein termed signal recognition particle (SRP) binds to its SP, see Figure 1. Subsequently, upon interaction of SRP with the SRP receptor at the ER membrane, SRP dissociates from SP which is then free to insert into the Sec61 channel. This pathway is named the co-translational, SRP-dependent pathway. In another route, the so-called post-translational pathway, the fully synthesised precursor is first freely released from the ribosome and then, supported by chaperones and/or SRP-independent machineries, enters the Sec channel. In *S. cerevisiae*, the Sec61 complex mediates both co- and posttranslational translocation whereas the mammalian Sec61 complex functions primarily on the co-translational pathway [3].

The eukaryotic Sec61 complex consists of three subunits, Sec61α, Sec61β, and Sec61γ. Sec61α, the central channel pore, is a multiple-membrane-spanning protein. Sec61β and Sec61γ are single-membrane-spanning proteins (Sbh1 and Sss1 in *S. cerevisiae*, respectively) (see Figure 1). Sec61α and its prokaryotic homologue SecY consist of two halves that form an hourglass-shaped pore with a constriction in the middle of the membrane, a plug domain in the luminal or extracellular cavity, and a lateral gate, which opens to the surrounding lipid phase [4,5]. The Sec61 complex in the ER membrane represents the major entry point for precursor polypeptides. Besides, the open Sec61 complex forms a Ca${}^{2+}$ -permeable channel. Furthermore, it was found that different human hereditary and tumour diseases are associated with Sec61 channel gating [6]. Interestingly, various human genetic diseases result from single point mutations in the SPs of certain precursor polypeptides [6,7,8].

## 2. Properties of Signal Peptides

**Figure 2:**
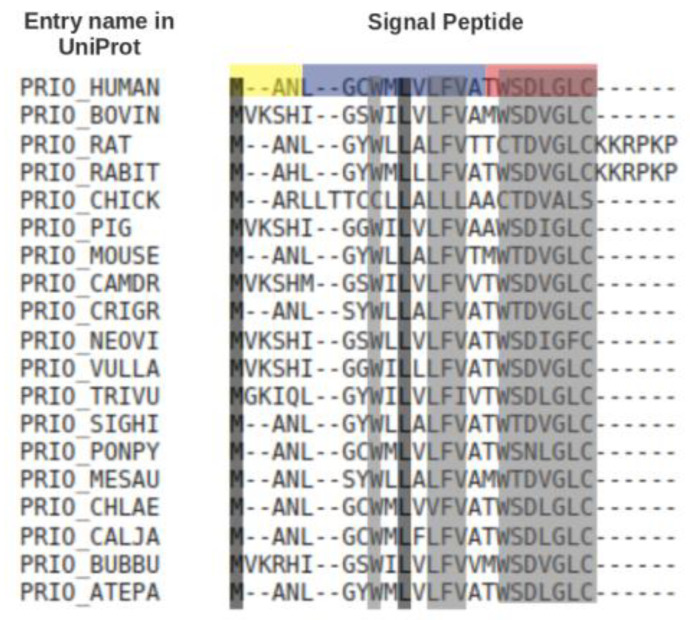
Multiple sequence alignment, produced by Kalign [21], of the signal peptides of the prion protein (PRNP) [22] from different organisms. For this, the signal peptide sequences of the prion protein from 68 organisms were collected from UniProt. CD-Hit [23] was used to remove sequences. with 100% sequence similarity. The 19 unique sequences(<100% sequence similarity) were used for the multiple sequence alignment. The alighnment was color-coded by conservation using the Sequence Manipulation Suite [24]. Residues that are identical among the sequences are given a black background, and those that are similar among the sequences are given a grey background. The N-, H- and C-regions of the human signal peptide, identified using Phobius [25], are highlighted in yellow, blue and red, respectively. A consensus sequence from the protein multiple sequence alignment is MANLxxWMLxLFVxxWSDVGLCxxxxxx.

Comparative sequence analysis showed that SPs have a typical length of ca. 20–30 residues, but may be up to 60 residues long [9]. They have a recognisable three-domain structure involving a net basic ‘N region’, a 7–13 residues long hydrophobic ‘H region’ and a slightly polar ‘C region’. Other than this, SPs of different precursor proteins have no noticeable sequence similarity to each other. In fact, SPs are often readily interchangeable, may tolerate a wide range of mutations and are capable of directing secretion in evolutionarily distant organisms. Figure 2 shows a multiple sequence alignment of the signal peptides of prion proteins from different organisms. Only two out of 21/27 positions are fully conserved, but eleven other positions are almost fully conserved.

In mammals, many signal sequences that promote successful targeting to the Sec61 complex cannot initiate translocation without further accessory membrane proteins such as TRAP or Sec62-63 being present at the site of translocation [5,10]. For example, the mechanistic role of the Sec62/63 complex is starting to be unveiled thanks to recent high-resolution cryo-EM structures of the heptameric Sec complex from *S. cerevisiae* [5,11,12,13], proteomic identification of putative Sec62- and Sec63-dependent precursor proteins [14], and molecular dynamics simulations [15]. The combination of such complementary techniques aids in solving the central puzzle of SP recognition by the Sec61 complex – how selectivity is achieved yet sequence diversity accommodated.

In water, functional SPs do not show substantial significant secondary structure [16]. Yet, in interfacial environments or in the specific environment of ribosome/SRP/translocon pore, e.g. when bound to SRP54-M from *M. jannaschii* [17], they have a high propensity for an α-helical conformation [16]. Their helical content is actually largest in the hydrophobic core of the signal sequence. For example, Yi and co-workers studied the conformation of a 25-residue-long functional SP of *E. coli* ribose binding protein using Circular Dichroism (CD) and Nuclear Magnetic Resonance (NMR) in solvents mimicking the amphiphilic environments around and in the Sec61 pore and compared this SP to a nonfunctional mutant-SP [18]. The functional peptide formed an 18-residue-long α-helix in a mixed 10% DMSO, 40% water, and 50% TFE solvent. The nonfunctional L9P mutant peptide did not have any secondary structure in that solvent but formed a 12-residue-long α-helix in 50% water: 50% TFE solvent. To correlate the α-helix-forming propensity of SPs with their translocation efficiency, Chi et al. determined the conformations of the SPs of two revertant ribose binding proteins in TFE/water solvent with CD and NMR spectroscopy [19]. In their CD experiments, both revertant SPs showed an intermediate helicity between those of wild-type and mutant SPs suggesting that the overall α-helical content is important for the functioning of SPs. Nguyen et al. reported that Gly/Pro amino acids are enriched in TRAP-dependent SPs [20]. Considering that Gly and Pro are known “helix breakers”, this observation suggests lower α-helical propensities of such SPs. In this sense, being a “weak” SP could be associated with having a weak α-helical propensity. Related to this, Schorr and co-workers found that Sec62/63-dependent SPs have an SP with a comparatively longer but less hydrophobic H-region and a less polar C-region [14].

## 3. Structural Studies of Eukaryotic Sec61:SP Complexes

**Figure 3:**
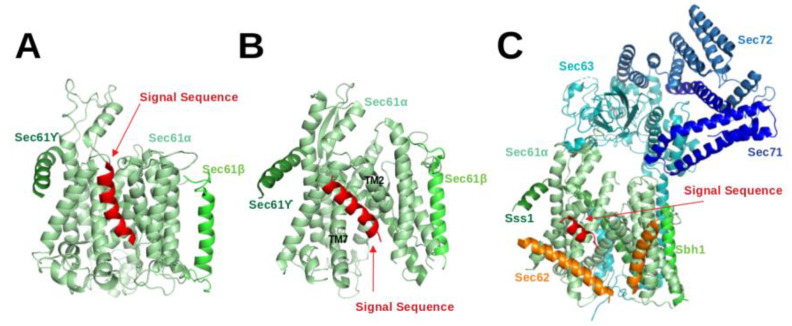
Shown are cryo-EM structures of the signal sequence-bound Sec61 complex (**A**) in the co-translational mode (4CG6; *Canis lupus*) [28], (**B**) in the co-translational mode (3JC2; *Canis lupus*) [4] and (**C**) in the post-translational mode (7AFT; *Saccharomyces cerevisiae*) [5].

The Sec61 complex consists of three subunits, namely α, β and γ. The α-subunit, Sec61α in eukaryotes, is the central component of the protein-conducting channel. Cryo-electron microscopy structures of the Sec61 complex have established its conformation in several functional states [4,5,11,12,13,26,27,28,29,30,31]. It contains ten transmembrane (TM) helices arranged in the central pore. The small β and γ subunits peripherally associate with the α-subunit and both of them have a single TM helix. In cryo-EM structures of Sec61 for the idle or inactive state, the helical plug domain, a helical structure formed in a sequence segment located immediately after TM1 of the α-subunit, is clearly visible inside the pore [27,29]. In translocating states, the plug moves away from its idle state position to allow transit of the SP [4,5,28]. Although its precise position could not be located, it is clear that the plug density was not present anymore in the pore. Recently, cryo-EM structures [4,5,28] demonstrated that the channel releases SPs to the lipid phase through the lateral-gate, a region between TM2 and TM7 of the α subunit (see Figure 3B).

Gogala et al. presented a cryo-EM structure of a mammalian ribosome-bound Sec61 complex from *Canis lupus familiaris* engaged in membrane insertion of nascent peptides (PDB entry 4CG6) [28]. The authors tested how hydrophobic and hydrophilic peptides translocate through the Sec61 channel on the example of hydrophilic (LepT) and hydrophobic (LepM) variants of the leader peptidase (Lep) protein. In the Sec61α complex engaged with the hydrophilic LepT peptide, the plug did not show a detectable shift compared to the idle state, the lateral-gate was partially opened (Figure 3 of [28]), and the lumenal part of the TM10 helix was shifted outward by ∼6 Å. The authors speculated that this shift of TM10 would be sufficient to provide the required opening for the accommodation of an extended translocating peptide segment between the plug and TM10. This would match previous crosslinking data showing that the translocating peptide is positioned near the plug helices, TM10 and TM5 of SecY (the α-subunit of prokaryotic SecYEG) [32]. However, Gogala et al. [28] were not able to model the location of the hydrophilic peptide within Sec61α in the electron density map. In contrast, the Sec61α complex engaged with the hydrophobic LepM peptide adopted a different conformation where the plug shifted compared to the idle state and the lateral-gate adopted an open conformation with displaced TM2 and TM7 helices. In that case, a rod-like extra electron density corresponding to three to four turns of an α-helix could be identified at the lateral-gate (4CG6) (see Figure 3A).

Later on, Voorhees et al. [4] presented a 3.6 Å resolution structure of the canine ribosome-Sec61 complex engaged with the secretory protein pre-prolactin (3JC2) (see Figure 3B). The signal sequence was again identified at the lateral-gate of Sec61α, similar to the previous hydrophobic peptide engaged structure (4CG6). Also here, the lateral-gate adopted an open conformation with the plug displaced from its idle position. The TM7 helix of Sec61α rotated as a rigid body relative to the plane of the membrane, thereby creating space between TM2 and TM7 for insertion of the SP (see Figure 3B, 3JC2). This cryo-EM structure demonstrated that the lateral-gate opening is asymmetric: the lumenal end of the gate shifts by a few Angstroms, whereas the cytosolic side remains closed. The authors compared the conformations of open/translocating state and idle state of Sec61α trying to explain how the SP reached this position. They suggested that the altered conformation of the lateral gate may result from a destabilized hydrogen bond network of the pore ring residues located in TM2, TM5, TM7 and TM10. Furthermore, they suggested that the interactions between the external hydrophilic surface of Sec61α and the hydrophobic lipid bilayer may also play an important role in the conformational dynamics of the lateral-gate. They did not notice any electron density of the plug domain inside the pore. Additionally, Voorhees et al. [4] reported that the density visible inside the ribosomal exit tunnel and in parts of the Sec61 channel suggests a looped configuration of the nascent chain, consistent with earlier crosslinking studies [33]. In both cryo-EM structures of mammalian ribosome-bound Sec61 complexes (co-translational mode), it was observed that hydrophobic SPs like to occupy the space between TM2 and TM7 and eventually insert into the lipid layer having a helical structure.

The abovementioned cryo-EM structures characterised the position of SPs in co-translational translocation. Recently, Weng et al. [5] presented a 4.4 Å resolution cryo-EM structure of the heptameric Sec complex from yeast (the Sec complex of yeast is consists of seven subunits, namely Sec61α, Sbh1, Sss1, Sec62, Sec63, Sec71, Sec72) bound to a substrate carrying the SP of prepro-α factor (see Figure 3C, 7AFT). As shown in Figure 1, the heptameric complex is formed only in the post-translational pathway. Three recent cryo-EM studies characterised the overall architecture of this heptameric post-translational translocon in other functional states without bound SPs [11,12,13]. Compared to the structures of the idle state [11], Sec61α adopted an even more open conformation with a relocated plug domain when a SP was located in the groove of the lateral gate of Sec61α [5]. The SP bound conformation of Sec61α is referred to as the translocating state. In that case, the SP adopts a α-helical conformation and appears at a similar position to those previously seen in co-translationally operating Sec61α [4] where SP is located close to TM7 and parallel to the TM2 helix of Sec61α. Its position is also fully consistent with previous cross-linking data that suggested that the prepro-α factor signal sequence localises near TM2 and TM7 of the Sec complex [34,35]. Furthermore, the SP is also nearby and oriented parallel to the TM2 helix of Sec62 which is consistent with previous chemical cross-linking data [35].

Despite this spectacular progress from structural biology, our molecular understanding of how substrates open the channel for translocation and how SPs interact specifically with the translocon is still incomplete.

## 4. Conformational Dynamics of the Sec61 Translocon and Bound SPs in Molecular Dynamics Simulations

**Figure 4:**
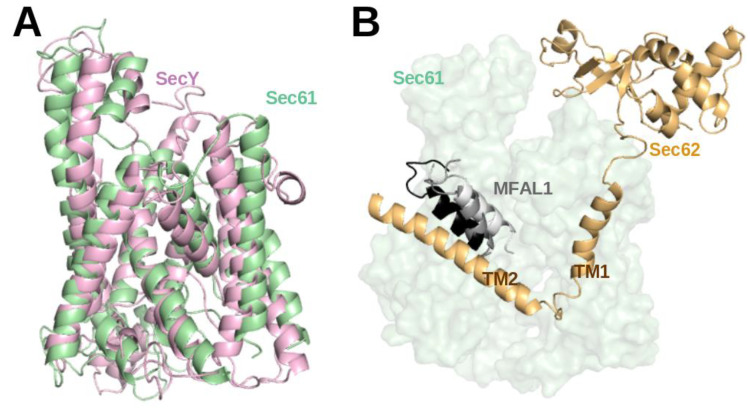
(**A**) Structural superposition of atomistic structures of SecY (light violet) and Sec61 (green). (**B**) Representative structures from a molecular dynamics simulation of SP bound Sec61 from yeast (unpublished). The conformations of the SPs at 0 ns, 500 ns, and 1 µs are coloured white, grey and black, respectively. For this, PDB entry 7AFT [5] detailing the cryo-EM structure of the signal sequence of Mating factor alpha-1 (MFAL1; UniProt ID: P01149) was used to model the SP (M1-A19) at the lateral-gate. The MD simulation was conducted in the presence of a part of Sec62 (orange) in a similar manner to [15].

Over the last few years, molecular dynamics (MD) studies provided detailed insight into co-translational and post-translational translocation. Since the structural changes of the translocon are essential for translocon-assisted protein insertion, many MD simulation studies focused on the conformational changes of the prokaryotic and eukaryotic Sec channels [15,36,37,38,39,40]. Furthermore, MD simulation studies explored how the Sec61 channel recognizes substrate peptides and directs them either to membrane insertion or translocation [41,42,43,44,45,46]. The contribution of thermodynamics and kinetics and the detailed role of the translocon in the exit mechanism were also explored by 2D and 3D coarse-grained simulations [47,48]. Initially, the structure of prokaryotic SecY (1RHZ, 3.5 Å) from 2004 [49] was used in many early simulation studies due to the lack of high-resolution structures for eukaryotic Sec61. When Voorhees et al. presented the first high-resolution structure of mammalian Sec61 in 2014 (3J7Q, 3.5 Å resolution) [29], it turned out that the structures of SecY and Sec61 channels are closely related (TM-align score 0.21 [50]) (see Figure 4A). This matches the facts that they share 53.6% sequence similarity, they are universally conserved protein-conducting channels and that they both either translocate proteins across or integrate them into the eukaryotic ER membrane and the prokaryotic plasma membrane [1].

Based on conformational analysis of the SecY channel using MD simulations, Gumbart et al. were able to explain the mechanism of lateral-gate opening, the roles of pore-ring and plug, and the effect of ribosome binding on the prokaryotic SecY channel [36,37,38]. Using steered MD simulations, they also pulled a deca-alanine peptide through the translocation pore of the archeal translocon and observed its consequences [46]. Due to the force exerted by the pulled deca-alanine, the plug was pushed out of the pore over a distance of 10 to 25 Å, allowing the deca-alanine to pass. Interestingly, this occurred without severe deformations of the helical structure of the peptide or the remainder of SecY. The pore ring expanded from its original size of ∼3.5–5.5 Å to a diameter of 7–12 Å. In contrast, a 19-residue long alanine/leucine helix (AL19) unfolded rapidly while being pulled through the pore in the same manner. Both deca-alanine and AL19 interacted strongly with the TM2 and TM7 helices of the translocon, suggesting this as another signal sequence recognition centre beside the lateral gate. Also, Tian and Andricioaei [51] mimicked the push-through of polypeptides across the translocon by pulling a virtual soft ball which demonstrated that the diameter of the SecY pore can be expanded to ∼16 Å without significant loss of its resilience.

Recently, Sun and coworkers [40] performed coarse-grained MD simulations of the mammalian structure of Sec61 to analyse its channel conformation. Their results suggest that the lateral gate is able to rapidly recover its partially-closed state after the nascent chain segment enters the bilayer. They also showed that the conformational dynamics of the lateral gate, pore ring and plug are interlinked. Moreover, Bhadra and Helms used all atomistic MD simulations to characterise conformational shifts in the Sec61 translocon from yeast due to the presence of the accessory protein Sec63 in the post-translational mode [15]. To collect representative statistics, they performed five independent simulations of 1 µs in length started either from the cryo-EM conformation of Sec61 bound to Sec63 or from uncomplexed Sec61. Their simulation results revealed that the wide pore opening is due to a reorientation of TM4 of Sec61 when it is attached to Sec63. This orientation shift of TM4 was governed by interactions between TM3 of Sec63 and TM1 of Sec61. Also, the simulations revealed that Sec63 affects the conformation of the lateral gate, and the plug moiety adopts different conformations within the channel in the presence and the absence of Sec63, respectively. Related to this finding, Mori et al. [39] proposed that the conformational transition from closed to pre-open states of SecY is associated with binding of SecA (an accessory protein in prokaryotes).

Apart from conformational changes of the translocon, several MD simulation studies also investigated the partitioning of SPs into the ER membrane. Zhang and Miller [45] explored the conformational landscape of the Sec translocon in the presence of hydrophobic (polyleucine, Leu30) versus hydrophilic (polyglutamine, Gln30) peptide substrates using coarse-grained enhanced sampling MD simulations of SecY. Based on the free energy cost of the structural fluctuations of the lateral gate, the authors concluded that a hydrophobic peptide substrate present in the translocon pore stabilises an open conformation of the lateral-gate, whereas a hydrophilic peptide substrate induces the closed conformation of the lateral gate. They reported that, for the hydrophilic substrate, the plug was preferentially positioned between the peptide substrate and the lateral gate, whereas for the hydrophobic substrate, the orientation was reversed such that the plug is behind the substrate with respect to the lateral gate. Thus, their results indicated that the hydrophobic substrate prefers an orientation where it is more exposed to the hydrophobic lipids of the membrane interior, whereas the hydrophilic substrate favours an orientation in which it remains fully inside the channel and shielded from the membrane by the plug.

To investigate how the hydrophobicity of SPs affects their insertion propensity into the membrane, Gumbart, Schulten and coworkers carried out all-atom MD simulations and umbrella sampling of different nascent transmembrane segments (a native Signal anchor, polyLeu, polySer, and polyGln) embedded in a ribosome-bound bacterial SecY translocon [52,53]. They tested the relationship between hydrophobicity of substrate and opening of the lateral-gate by performing all-atom classical MD simulations starting from different SecY conformations (closed-state, open-state and intermediate state). They observed that the degree of opening of the SecY lateral gate apparently did not depend on the hydrophobicity of the nascent chain inside the channel, at least not on the 1–2 µs time scale. Yet, the SP was able to spontaneously move into the membrane or back into the channel, depending on its hydrophobicity. Hence, the interactions between SP and hydrophobic lipid likely play an important role for the insertion of SPs into the membrane. Zhang and Miller [47] also investigated the role of the hydrophobicity of single anchor peptides on their insertion mechanism by coarse-graining the ribosome–nascent chain–membrane system and representing it in two spatial dimensions. They found that particular salt-bridge contacts between the nascent-protein N-terminus, cytosolic translocon residues, and phospholipid head groups favoured conformations of the nascent protein chain consistent with the type II topology. A type II topology refers to single-pass transmembrane proteins having an extracellular (or luminal) C-terminus and cytoplasmic N-terminus. In contrast, increasing the SP hydrophobicity stabilised nascent-protein configurations consistent with the type III topology [48]. A Type III topology membrane proteins have their N-terminal domains targeted to the ER lumen. Their detailed simulation studies provided a mechanistic basis for understanding experimentally observed correlations between the topology of integral membrane proteins and their amino-acid sequence. Recently, Niesen and coworkers [43] presented a novel coarse-grained model for co-translational membrane protein integration via the Sec translocon that enables simulation of long time-scales relevant for protein biosynthesis. The model was parameterised to reproduce sequence-specific NC-translocon interactions and enabled simulating practically any nascent peptide chain by providing only its amino-acid sequence information as input. They showed that a more pronounced hydrophobicity of the signal anchor increases the probability of membrane integration. Their simulation results also demonstrated that increasing the length of the C-terminal loop of the TM segment raises the probability that the TM segment adopts a type II topology (N${}_{cyt}$/C${}_{ER}$). Their studies helped to elucidate the effects of sequence, translation rate, and external forces on the probability of nascent-chain integration into the membrane and to rationalise the resulting orientation of TMDs with respect to the membrane [44]. Besides, by combining site-directed mutagenesis in the yeast Sec61 translocon (in vivo) and MD simulations of SecY, it was shown that the membrane insertion propensity of signal-anchor sequences strongly depends on the positions of hydrophobic residues in the sequence segment and on its interaction with the six pore ring residues [41]. Furthermore, Rychkova and Warshel presented quantitative insertion free-energy profiles where they investigated the effect of SP and translocon mutations on the insertion free-energy using a coarse-grained model of the SecY translocon [42]. Their systematic analysis of the free-energy profile revealed that increasing the positive charge in the N-terminus increases the fraction of C-translocated peptides (N${}_{cyt}$/C${}_{ER}$, type II topology), whereas increasing the length of the helix reduced this fraction.

The role of water molecules in protein translocation via SecY was also investigated using all-atomistic MD simulations [54]. This study showed that the water molecules in the translocon pore do not behave as in bulk phase since they exhibited anomalous diffusion, had highly retarded rotational dynamics, and aligned their dipoles along the SecY The authors suggested that the water molecules facilitate the interaction between lipids and a peptide located inside the SecY and the dipole alignment of water molecules along the SecY axis may crucially affect the interaction of the positively charged N-terminus of a signal sequence with the translocon and may hence support membrane integration.

Recently, a structure of the active post-translational Sec complex from *Saccharomyces cerevisiae* bound to a SP was determined by cryo-electron microscopy [5]. We performed a 1 µs long atomistic MD simulation of this structure whereby the 13 amino-acid long SP of Mating factor alpha-1 (MFAL1) was modelled at the lateral gate. Interestingly, the SP shifted toward the TM2 helix of Sec62 (see Figure 4B) during the 1 µs simulation (unpublished). This hints at a direct interaction between SP and Sec62 which is in agreement with previous chemical cross-linking data [35]. Recently, Itskanov et al. suggested based on atomistic MD simulations that the presence of Sec62 also prevents lipids from invading the channel pore through the open lateral gate [13].

## 5. Molecular Docking Helps in Understanding the Interaction between Signal Peptide and Sec61 Translocon

Molecular docking is a well-known computational approach to investigate or predict the potential interactions between two molecules [55]. Our extensive MD simulations mentioned before [15] revealed that TM2, TM4, TM7 and the plug of Sec61 adopt different conformations in both Sec63-bound and free states. After observing these conformational transitions, we wanted to check whether those Sec61 structural elements affected by Sec63 are indeed related to substrate translocation across the membrane. To this aim, we performed molecular docking of flexible SPs in the translocon pore that was treated as rigid protein using the ADCP docking tool [56] from the Autodock developers that was optimised for docking of flexible peptides. Docking indeed showed that the hydrophobic core of the signal anchors of SRP-dependent substrates has a higher propensity for the volume between the C-terminus of TM2 and the N-terminus of TM7 than the hydrophobic core of SPs of SRP-independent substrates [15]. This suggests that the translocation process depends on the interaction of targeting sequences with the lateral gate which is in good agreement with experimental findings [57].

**Figure 5:**
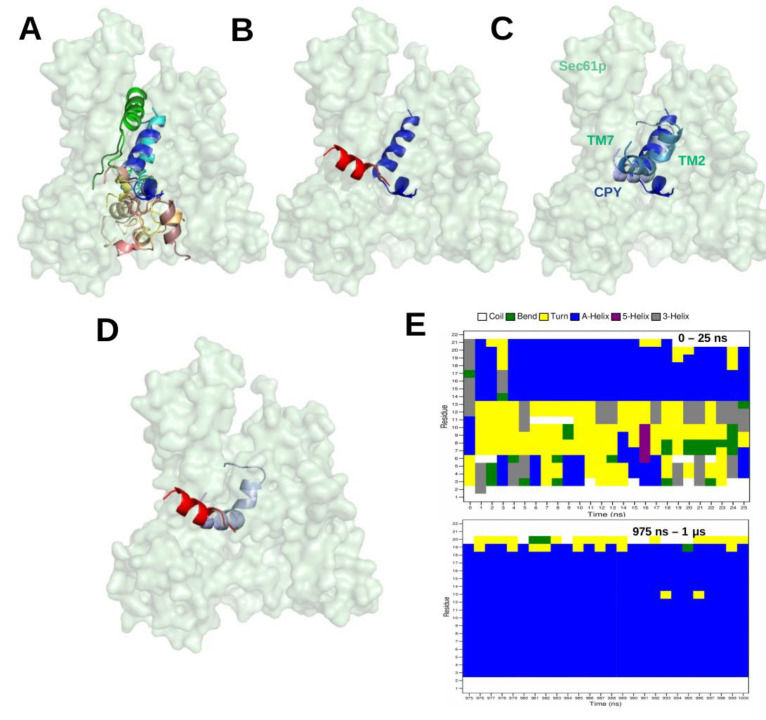
(**A**) The top ten ranked docking positions of the signal peptide (SP) of *Carboxypeptidase Y* (CPY) inside the Sec61 pore from yeast in its conformation when bound to Sec62 and Sec63 in the Sec complex. The ADCP software [56] was used for molecular docking [15] (**B**) Structural superimposition of the most favourable docking pose with lowest binding energy (3 conformations out of 10 (blue variant in (**A**)) and of MFAL1-SP in the cryo-EM structure (red representing 7AFT) (**C**) The most favourable docking position (blue) was used as starting structure for a subsequent MD simulation. The conformations of the SP at 0 ns, 500 ns, and 1 μs from the MD simulation trajectory are represented in blue, skyblue and lightblue, respectively. (**D**) Structural superimposition of CPY-SP in the final snapshot (1μs) of the MD simulation (lightclue; CPY) and of MFAL1-SP in the cryo-EM structure (red, 7AFT). (**E**) DSSP [59] profiles of the signal peptide of Carboxypeptidase Y obtained from the first 25 ns and last 25 ns (975 ns–1μs) of the simulation.

This study was a first attempt to use molecular docking to characterize the binding modes of SPs. Since the developers of the docking tool ADCP recommended its use only for peptides with a maximal length of 20 residues [56], only relatively short SPs can be tested in this way. The SP of *Carboxypeptidase Y* (CPY) is such a short SP with a length of only 21 amino acids. Hence, we performed several docking [15] runs for this SP using the Sec61 conformation when it is bound to the Sec62-Sec63 complex, which is believed to reflect its conformation during post-translational protein translocation. Figure 5A shows the top-ten ranked docking positions of the CPY-SP in the channel pore. However, the most favourable conformation of the docked CPY signal peptide differs somehow from the cryo-EM structure; 7AFT (Figure 5B). As a test we performed a single 1 microsecond-long MD simulation starting from the lowest-energy docking pose. The technical details of the MD protocol match those in [15]. In the MD simulation, the N-terminus of CPY-SP shifted from TM2 to TM7 (see Figure 5C), and the SP gradually shifted to occupy a very similar position as in the cryo-EM structure, 7AFT (see Figure 5D).Interestingly, over time, the SP conformation adopts an increasingly larger portion of alpha-helical conformation during the MD simulation ( Figure 5E) which is in agreement with the cryo-EM structure. Figure 5E suggests that the region 13–21 a.a. of SP remains alpha-helical throughout the MD simulation, whereas, the region 3–12 a.a. of SP gradually adopts α-helical conformation. Although the MD simulation results have not been published so far, we like to add this study here as an example where docking was used in combination with MD simulations.

The MD simulation results suggest that the combination of docking and MD simulation is a suitable approach to characterise signal peptide recognition and conformational dynamics of the signal peptide bound to Sec61. Recently, the combined approach was used, for example, to provide valuable insight into drug discovery [58]. Docking protocols generally do not consider conformational flexibility of the receptor (here, Sec61 translocon), presence of water and lipids what may affect the reliability of the resulting docking poses. In contrast, MD simulations can treat both ligand and protein as flexible and the effects of explicit water molecules and lipid are directly captured. However, MD simulations are very time-consuming and can get trapped in local minima. Therefore, combining the two techniques in one protocol, where docking generates the most favorable conformation of the complex (lowest binding energy and largest cluster size) and MD simulation is then applied to explore the effect of flexibility of the molecules, water and lipid, could be an efficient approach to understand the conformation of SPs within the channel pore. Moreover, it would be appropriate to collect representative statistics from several independent MD simulations started with different starting velocities or from different docking positions.

## 6. Conclusions

The computational studies reviewed here illustrate the important contributions made by molecular dynamics simulations to the aim of better understanding Sec-SP dynamics and energetics during translocation. Application of these methods also enables us to discover mechanistic details how SP characteristics and allosteric effectors such as the accessory proteins (Sec62 and Sec63) influence protein translocation mediated by the Sec61 complex. Molecular simulations also help in connecting aspects of translation kinetics to the conformational sampling of peptide chains during membrane insertion or translocation to the ER. Thus, simulations of such systems help explain a number of experimental observations and can also serve as a catalyst to motivate new experimental studies. However, several open issues remain unanswered to date. How does the interaction of SPs and the Sec61 complex (except the lateral gate region which has been widely studied) help in protein translocation? What are the conformations of those parts of the Sec complex that are missing in the cryo-EM structures? Experimentally it has been noticed that some of these parts are essential for protein translocation (as an example, C-terminus of Sec63, N-terminus of Sec62 and N-terminus of Sec61 etc.) [3,60]. How do those parts interact with SPs? Thus, it can be expected that molecular simulations in combination with complementary experimental techniques will continue to explore new avenues in order to offer new insights into the complex behaviour of the nascent proteome and how this is influenced by translation dynamics.
